# A Method for the Isolation and Characterization of Mycosporine-Like Amino Acids from Cyanobacteria

**DOI:** 10.3390/mps1040046

**Published:** 2018-12-03

**Authors:** Siripat Ngoennet, Yasuhiro Nishikawa, Takashi Hibino, Rungaroon Waditee-Sirisattha, Hakuto Kageyama

**Affiliations:** 1 Department of Microbiology, Faculty of Science, Chulalongkorn University, Payathai Road, Pathumwan, Bangkok 10330, Thailand; siripat.ngoennet@gmail.com; 2 Faculty of Pharmacy, Meijo University, 150 Yagotoyama, Tenpaku-ku, Nagoya, Aichi 468-8503, Japan; yasuhiro@meijo-u.ac.jp; 3 Faculty of Science and Technology, Meijo University, 1-501 Shiogamaguchi, Tenpaku-ku, Nagoya, Aichi 468-8502, Japan; hibino@meijo-u.ac.jp

**Keywords:** mycosporine-like amino acids, mycosporine-2-glycine, shinorine, porphyra-334

## Abstract

This report provides a broadly applicable and cost-effective method for the purification of mycosporine-like amino acids (MAAs) from cyanobacteria. As MAAs are known to have multiple bioactivities for health and beauty, a universal isolation method of MAAs from biomass is attractive. In particular, the biomass of photosynthetic microorganisms such as cyanobacteria is of interest as a natural source of useful compound production, because of their photoautotrophic property. The method presented here is applicable for the isolation of mycosporine-2-glycine (M2G), which is a rare MAA produced in a halotolerant cyanobacterium. This method also allowed for the isolation of two of the most common MAAs, shinorine (SHI) and porphyra-334 (P334). A three-step separation process using low pressure liquid chromatography yielded purified MAAs, which were characterized by nuclear magnetic resonance (NMR) and liquid chromatography-mass spectrometry (LC/MS) analyses. The purified MAAs exhibited free radical scavenging activity in the 2,2′-azino-*bis*(3-ethylbenzothiazoline-6-sulfonic acid) (ABTS) assay. The experimental parameters obtained in this report may allow for a scale-up of the MAA purification process for future industrial applications.

## 1. Introduction

Cyanobacteria are Gram-negative prokaryotic microorganisms that can convert solar energy and CO_2_ into chemicals by photosynthesis. Because of their photoautotrophic property, higher growth rate, and genetic manipulability, cyanobacteria have gained much attention as a promising group of microorganisms capable of producing industrially important compounds. Mycosporine-like amino acids (MAAs) are water-soluble low molecular weight (<400 Da) secondary metabolites, which are synthesized in various organisms, such as cyanobacteria and other prokaryotes, eukaryotic micro-organisms, marine green and red macroalgae, corals, and terrestrial lichens [1]. The maximum absorptions of MAAs are in the ultraviolet (UV) region (ranging from 268 to 362 nm) with high molar extinction coefficients depending on their molecular structure [1]. Although the physiological roles of MAAs in cyanobacteria have not been fully elucidated, MAAs are known as multifunctional compounds [2]. MAAs have been commercialized as the sunscreen reagent Helioguard^®^365, which contains the liposomal MAAs, shinorine (SHI) and porphyra-334 (P334). Helioguard^®^365 is available in the global market [3]. In addition to their UV-absorbing property, it has been reported that MAAs possess additional biological functions. For instance, mycosporine-2-glycine (M2G), which was isolated from the halotolerant cyanobacterium *Aphanothece halophytica*, exhibited free radical scavenging activity [4], protection against oxidative stress-induced cell death [4], osmoprotectant activity [5], the inhibition of collagenase activity [6,7], and the inhibition of protein glycation [7].

The generation of pure fractions of MAAs is crucial for elucidating their biological functions. To date, a broadly applicable method for the extraction and characterization of MAAs in cyanobacteria has been reported [8]. However, this study did not describe a MAA purification strategy. In contrast, there have been many reports describing the MAA purification methods from cyanobacteria based on high performance liquid chromatography (HPLC) systems [2,9,10,11]. Techniques utilizing analytical HPLC for the purification of MAAs are insufficient for generating a sufficient amount of MAAs for the assessment of their biological activities. Furthermore, preparative HPLC is a rather expensive technique [12]. Therefore, the development of a versatile and simple preparative purification process that is cost-effective is desirable. Here, we established a three-step separation method using low pressure liquid chromatography that effectively purified M2G from *A. halophytica*. Moreover, the liposomal MAAs SHI and P334, which are present in the sunscreen reagent Helioguard^®^365, were also purified with this method. An nuclear magnetic resonance (NMR) analysis indicated that our method generated highly pure fractions of MAAs. In addition, the purified MAAs exhibited free radical scavenging activity demonstrated via the 2,2′-azino-*bis*(3-ethylbenzothiazoline-6-sulfonic acid) (ABTS) assay. Taking into account these observations, our purification method is applicable for the isolation of various MAAs produced in cyanobacteria.

## 2. Experimental Design

**Figure 1:**
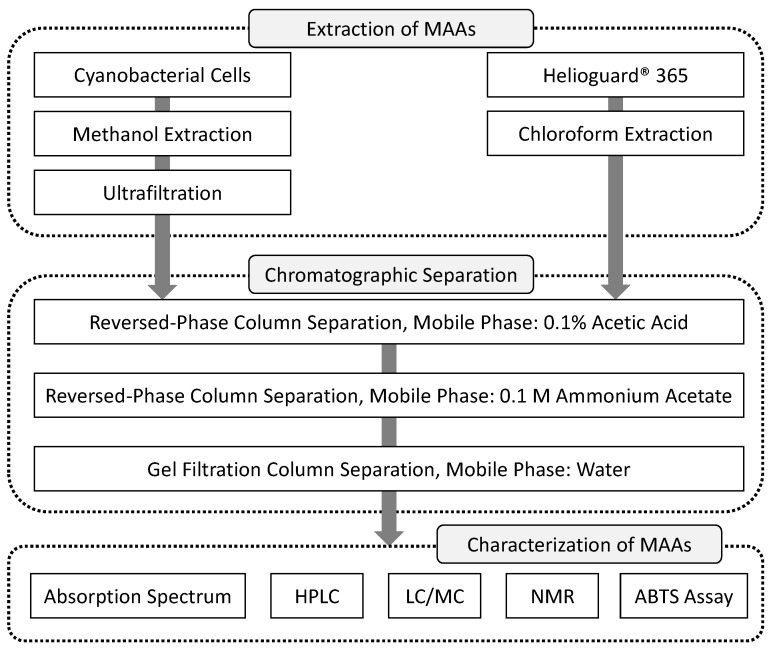
Stages of the isolation and characterization of mycosporine-like amino acids (MAAs).

Our method consists of the following three stages: (1) extraction, (2) separation (purification), and (3) characterization, as shown in Figure 1.

In the extraction stage, M2G was extracted from cyanobacterial cells utilizing methanol. The addition of methanol and following sonication treatment could disrupt the cells, and released M2G was dissolved in methanol. On the other hand, the MAAs SHI and P334 were extracted from Helioguard^®^365 utilizing chloroform, which has a weaker polarity than methanol, because these MAAs were liposomal form in Helioguard^®^365. After the disruption of liposome, the MAAs moved to the aqueous phase.

In the chromatographic separation stage, a two-step reversed-phase chromatography strategy was performed using acidic and neutral mobile phases designed to remove the impurities from the target MAAs. The last step of the chromatographic separation stage consisted of gel filtration chromatography that further improved the purity of the MAAs and allowed for the exchange from the solvent to water.

The MAAs were characterized by the following analyses: absorption spectra, analytical HPLC, liquid chromatography-mass spectrometry (LC/MS), and NMR. Moreover, the ABTS assay was used to test whether the purified MAAs exhibited antioxidant activity.

### 2.1. Materials

#### 2.1.1. Extraction of MAAs

- Cyanobacterial cells (cyanobacterial cells were available from culture collections, such as the American Type Culture Collection (ATCC), Manassas, VA, USA).
- Helioguard^®^365 (Mibelle Biochemistry, Buchs, Switzerland)
- HPLC grade methanol (Wako Pure Chemicals Industries, Osaka, Japan; Cat. no. 132-06471)
- Chloroform (Kanto Chemical, Tokyo, Japan; Cat. no. 07278-00)
- Amicon Ultra-4 Ultracel-3K (Merck Millipore, Darmstadt, Germany; Cat. no. UFC800396)

#### 2.1.2. Purification of MAAs

- Acetic acid (Kanto Chemical, Tokyo, Japan; Cat. no. 01021-00)
- Ammonium acetate (Sigma-Aldrich, Tokyo, Japan; Cat. no. 01-4390-5)
- Ethanol (Sigma-Aldrich, Tokyo, Japan; Cat. no. 09-0770-4)
- Nanosep Membrane Filter, 0.45 µm (Pall Life Sciences, MI, USA; Cat. no. ODGHPC34)
- Cosmosil 40C_18_-PREP (Nacalai Tesque, Kyoto, Japan; Cat. no. 37932-86)
- Sephadex G-10 (GE Healthcare, Uppsala, Sweden; Cat. no. 51185200-EG)
- Polycarbonate chromatography column (18 × 300 mm) (Eyela, Tokyo, Japan; Cat. no. 166130)
- Polycarbonate chromatography column (14 × 500 mm) (Eyela, Tokyo, Japan; Cat. no. 166090)

#### 2.1.3. Characterization of MAAs

##### LC/MS Analysis

- Methanol for LC/MS (Kanto Chemical, Tokyo, Japan; Cat. no. 25185-79)
- APCI Positive Calibration Solution for the AB Sciex Triple TOF system (AB SCIEX, Framingham, MA, USA; Cat. no. 4460131)
- Triart C18 column (2.1 × 33 mm) (YMC, Kyoto, Japan; Cat. no. TA12S03-H3Q1PTH)

##### NMR Analysis

- Methanol-*d*_4_, for NMR (Acros Organics, NJ, USA; Cat. no. 351460075)

##### ABTS Assay

- ABTS (Tokyo Chemical Industry, Tokyo, Japan; Cat. no. A2166)
- Potassium peroxodisulfate (Sigma-Aldrich, Tokyo, Japan; Cat. no. 24-5220-2)
- 6-Hydroxy-1.5.7.8-tetramethyl-chroman-2-carboxylic acid (Trolox) (Tokyo Chemical Industry, Tokyo, Japan; Cat. no. H0726)

### 2.2. Equipment

#### 2.2.1. Extraction of MAAs

- Sonicator VP-5s (Taitec, Tokyo, Japan)
- Rotary evaporator VC-15s (Taitec, Tokyo, Japan)

#### 2.2.2. Purification of MAAs

- Low pressure liquid chromatography system AKTA prime (Amersham Pharmacia Biotech, NJ, USA)
- Freeze dryer FDS-1000 (Eyela, Tokyo, Japan)
- Photospectrometer BioSpec-nano (Shimadzu, Kyoto, Japan)

#### 2.2.3. Characterization of MAAs

##### Absorption Spectra Analysis

- Photospectrometer BioSpec-nano (Shimadzu, Kyoto, Japan)

##### Analytical HPLC Analysis

- Analytical HPLC L-2000 system (Hitachi High Technologies, Tokyo, Japan)

##### LC/MS Analysis

- LC/MS Triple TOF 6600 system (AB SCIEX, Framingham, MA, USA) along with the Nexera XR system (Shimadzu, Kyoto, Japan)

##### NMR Analysis

- AVANCE III HD 600 NMR spectrometer equipped with a CryoProbe Prodigy (Bruker, Rheinstetten, Germany)
- NMR test tube HG-type (Wako Pure Chemical Industries, Osaka, Japan)

##### ABTS Assay

- Photospectrometer BioSpec-nano (Shimadzu, Kyoto, Japan)

## 3. Procedure

### 3.1. Extraction of MAAs (Time of Completion: 5–6 h for M2G, 2–3 h for SHI and P334)

#### 3.1.1. Extraction of M2G from *A. halophytica* Cells

1. Collect *A. halophytica* cells from liquid cultures. For the induction of M2G bioproduction in *A. halophytica*, use a BG-11 liquid medium plus Turk Island salt solution, which contains 2.5 M NaCl. Typically, ~3 g fresh weight cells are obtained from a 500 mL culture from cells in the stationary growth phase.
2. Add methanol and resuspend the cells. The volume of methanol was determined with the following formula: methanol volume [mL] = cell fresh weight [g] × 8.
3. Disrupt the cells with a sonicator. The suspension should be cooled on ice during sonication. Typical parameters for disrupting ~3 g fresh weight cells using the VP-5s instrument comprise the following: output (7), on time (30 s), off time (30 s), and total on time (60 s).
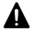
**CRITICAL STEP** The cells should be completely disrupted.
4. Allow the suspension to settle at room temperature for 15 min.
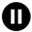
**PAUSE STEP** The suspension can be stored at 4 °C overnight.
5. Centrifuge the suspension at 15,000 × *g* for 10 min at 25 °C.
6. Transfer the supernatant to a new tube.
7. Desiccate the supernatant with a rotary evaporator at room temperature. This step takes approximately 2 h.
8. Dissolve the dried material in 4.0 mL of water.
9. Remove the undissolved compounds by centrifugation at 15,000 × *g* for 10 min at 25 °C.
10. Add 80 µL of chloroform to the supernatant, and shake the tube. In this step, the pigments move to the chloroform phase. With the addition of this amount of chloroform, the color of the solution turns from deep green to clear.
11. Centrifuge the samples at 15,000 × *g* for 10 min at 25 °C.
12. Transfer the supernatant (aqueous phase) to an Amicon Ultra-4 Ultracel-3K Centrifugal Filter.
13. Centrifuge the filter at 15,000 × *g* at 25 °C until most of the solution is passed through the membrane. Collect the flow through fraction. This step can remove high molecular weight compounds and takes approximately 1–2 h. The samples should be stored at 4 °C until they are subjected to the separation step.

#### 3.1.2. Extraction of SHI and P334 from Helioguard^®^365

1. Concentrate 2.0 mL of Helioguard^®^365 to a volume of ~200 µL with a rotary evaporator. This step takes approximately 1–2 h.
2. Dissolve the concentrated sample in 1.0 mL of water.
3. Remove the undissolved compounds by centrifugation at 15,000 × *g* for 10 min at 25 °C.
4. Add 1.0 mL of chloroform to the supernatant, and mix vigorously. Chloroform can disrupt the liposomal forms of SHI and P334. In this step, the MAAs move to the equal amount of the aqueous phase.
5. Centrifuge the samples at 15,000 × *g* for 10 min at 25 °C.
6. Transfer the upper phase (aqueous phase) into a new tube.

### 3.2. Preparation of Columns (Time of Completion: 2–3 h)

#### 3.2.1. Preparation of Reversed-Phase Column

1. Open the cap of the empty polycarbonate chromatography column (18 mm × 300 mm) and place it in a vertical position.
2. Pour Cosmosil 40C_18_-PREP resin into the column until it is completely filled with the resin.
3. Close the cap, and connect the column to a low-pressure liquid chromatography system.
4. Apply at least three column volumes of ethanol to the column.

#### 3.2.2. Preparation of Gel Filtration Column

1. Open the cap of the empty polycarbonate chromatography column (14 mm × 500 mm) and place it in a vertical position.
2. Soak Sephadex G-10 resin in water for at least 30 min.
3. Pour the resin into the column and allow it to settle using gravity. Repeat this step until the column is completely filled with the resin.
4. Close the cap, and connect the column to a low-pressure liquid chromatography system.
5. Apply at least two column volumes of water to the column.

### 3.3. Purification of MAAs with a Three-Step Chromatographic Separation (Time of Completion: 3–4 Days)

All separation steps are conducted at room temperature.

#### 3.3.1. Separation by Reversed-Phase Chromatography Using 1% Acetic Acid (*v*/*v*) as a Mobile Phase

##### Preparation of Samples

Before applying the samples to the column, add acetic acid to the MAA samples to set the final concentration to 1% (*v*/*v*).
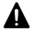
**CRITICAL STEP** Pass the sample through a filter (pore size: 0.45 µm) if the solution contains insoluble compounds.

##### Separation

1. Before separation, apply at least one column volume (~80 mL) of 1% acetic acid in water to the column.
2. After the equilibration of the column, inject the sample (~4 mL for M2G extracted from *A. halophytica* cells; ~1 mL for SHI and P334 extracted from Helioguard^®^365) into the column.
3. Apply 1% acetic acid to the column at a flow rate of 3.0 mL/min, and collect 2.0 mL fractions.
4. Measure the absorption of the collected fractions at 330 nm with a photospectrometer. The eluted peaks of M2G, SHI, and P334 were typically found in fractions #38, #35, and #50, respectively.
5. After separation, wash the column with 25 mL of 96% ethanol.
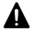
**CRITICAL STEP** Do not use methanol or other organic solvents if a polycarbonate column was used.
6. Combine the fractions containing each MAA, and lyophilize them.

#### 3.3.2. Separation by Reversed-Phase Chromatography Using 0.1 M Ammonium Acetate as a Mobile Phase

##### Preparation of Samples

Dissolve the lyophilized samples in 4.0 mL of 0.1 M ammonium acetate.
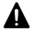
**CRITICAL STEP** Pass the sample through a filter (pore size: 0.45 µm) if the solution contains insoluble compounds.

##### Separation

1. Before separation, apply at least one column volume (~80 mL) of 0.1 M ammonium acetate to the column.
2. After the equilibration of the column, inject the sample (~4 mL) into the column.
3. Apply 0.1 M ammonium acetate to the column at a flow rate of 3.0 mL/min and collect 2.0 mL fractions.
4. Measure the absorption of the collected fractions at 330 nm with a photospectrometer. The eluted peaks of M2G, SHI, and P334 are typically found in fractions #36, #33, and #50, respectively.
5. After separation, wash the column with 25 mL of 96% ethanol.
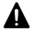
**CRITICAL STEP** Do not use methanol or other organic solvents if a polycarbonate column was used.
6. Combine the fractions containing each MAA, and lyophilize them.

#### 3.3.3. Separation by Gel Filtration Chromatography

##### Preparation of Samples

Dissolve the lyophilized samples in 4.0 mL of water.


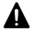
**CRITICAL STEP** Pass the sample through a filter (pore size: 0.45 µm) if the solution contains insoluble compounds.

##### Separation

1. Before separation, apply at least one column volume (~80 mL) of water to the column.
2. After the equilibration of the column, inject the sample (~4 mL) into the column.
3. Apply water to the column at a flow rate of 2.0 mL/min, and collect 2.0 mL fractions.
4. Measure the absorption of the collected fractions at 330 nm with a photospectrometer. The eluted peaks of M2G, SHI, and P334 were typically found in fractions #18–19.
5. After separation, wash the column with 80 mL of water.
6. Combine the fractions containing each MAA, and lyophilize them.
7. The quantitation of purified MAAs were determined using the following molar extinction coefficients: SHI (44,668 M^−1^ cm^−1^) and P334 (42,300 M^−1^ cm^−1^). The molar extinction coefficient of M2G was assumed to be identical to that of the structurally similar compound SHI, as reported previously [13].

### 3.4. Characterization of MAAs (Time of Completion: 1–2 Days)

#### 3.4.1. Absorption Spectra Analysis

**Figure 2:**
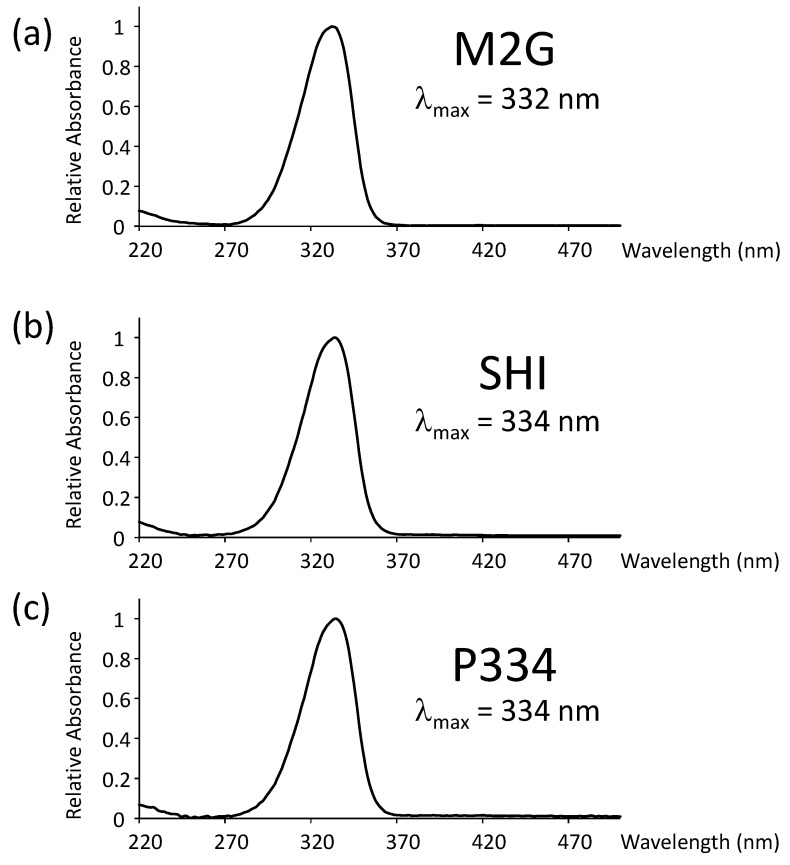
Absorption spectra of (**a**) mycosporine-2-glycine (M2G), (**b**) shinorine (SHI), and (**c**) porphyra-334 (P334).

Measure the absorption spectra with a photospectrometer. The spectra of target MAAs obtained in this study are shown in Figure 2.

#### 3.4.2. Analytical HPLC

1. Analyze the MAAs using an analytical HPLC instrument. All of the MAAs examined in this study can be separated with a typical octadecylsilyl (ODS) column. The separation conditions in this study comprised the following:
  Columns: Shim-pack FC-ODS reversed-phase column (3 µm; 150 × 4.6 mm; Shimadzu, Kyoto Japan) connected to a guard column (30 × 4.6 mm) that contained the same packing material as the main column.
  Injection volume: 10 µL
  Mobile phase: 1% acetic acid (*v*/*v*) in water
  Flow rate: 0.4 mL/min
  Separation temperature: 35 °C
  Detection: 330 nm using a UV-visible detector
2. If the authentic standards of MAAs are utilized, compare the retention times of the target MAAs to the standards.

#### 3.4.3. LC/MS

1. Prepare a 200 nM solution of MAAs by dissolving them in 10% methanol (*v*/*v*) in water.
2. Mass calibration was conducted using the APCI Positive Calibration Solution (AB Sciex). The conditions for both HPLC and MS comprised the following:
  [HPLC] Nexera XR system (Shimadzu)
  Columns: Triart C18 column (3 µm; 33 × 2.1 mm) (YMC, Kyoto, Japan)
  Injection volume: 2 µL
  Mobile phase: 10% methanol in water (0 min) to 70% methanol in water (3 min) to 100% methanol (7 min); linear gradient
  Flow rate: 0.3 mL/min
  Separation temperature: 35 °C
  [MS] Triple TOF 6600 system
  Source housing: DuoSpray ion source
  Ionization: ESI positive ion mode
  Source temperature: 500 °C
  Experimental type: TOF MS

#### 3.4.4. NMR

1. Dissolve the samples (~600 µg) in 0.6 mL of methanol-*d*_4_.
2. Transfer the resulting solution to an NMR test tube.
3. Measure the NMR with an AVANCE III HD 600 spectrometer.
4. Chemical shift values are reported using residual CD_2_HOD (δH 3.31) and CD_3_OD (δC 49.15) for the references. The ^1^H-NMR spectra are reported as follows: δ (number of protons, multiplicity, coupling constant J Hz). The multiplicities are indicated by s (singlet), d (doublet), and ABq (AB quartet).

#### 3.4.5. ABTS Assay

1. Prepare 7 mM ABTS and 2.45 mM potassium persulfate by dissolving in water.
2. Mix the 7 mM ABTS solution with the 2.45 mM potassium persulfate solution (1/1, *v*/*v*), and allow the mixture to settle for 12–16 h at 25 °C in the dark. During this incubation, the ABTS radical cation (ABTS^•+^) is produced.
3. Dilute the ABTS^•+^ solution with ethanol to an absorption of approximately 0.70 at 413 nm.
4. Mix 18 µL of the diluted ABTS^•+^ solution with 2 µL of the test samples. In this study, Trolox was used as a positive control.
5. Incubate the mixtures at 25 °C for 15 min and measure the absorption at 413 nm.
6. Determine the % inhibition of the ABTS radical using the following equation: inhibition % = ((*A*b − *A*t)/*A*b) × 100, where *A*b and *A*t are the absorbances of the blank and tested samples, respectively.

## 4. Expected Results

Typical purification summaries of M2G from *A. halophytica*, as well as SHI and P334 from Helioguard^®^365 are shown in Table 1.

**Figure 3:**
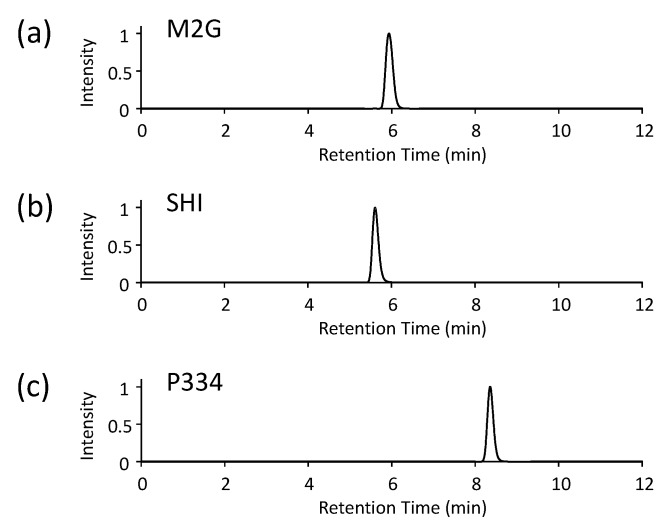
Analytical high performance liquid chromatography (HPLC) profiles of (**a**) M2G, (**b**) SHI, and (**c**) P334. The retention times of M2G, SHI, and P334 were 5.9, 5.6, and 8.4 min, respectively.

The absorption spectra and analytical HPLC chromatograms of M2G, SHI, and P334 are shown in Figure 2 and Figure 3, respectively. The maximum absorption wavelengths of M2G, SHI, and P334 were 332, 334, and 334 nm, respectively, as previously reported [14,15]. The molecular masses of these MAAs were confirmed by LC/MS analysis. The mass spectra revealed that the molecular masses of M2G, SHI, and P334 were 302 Da ([M + H]^+^ at *m/z* 303), 332 Da ([M + H]^+^ at *m/z* 333), and 346 Da ([M + H]^+^ at *m/z* 347), respectively.

The molecular structures of these purified MAAs were confirmed by NMR analysis. The NMR data is shown below.



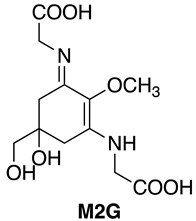



**M2G**: **^1^H NMR** (600 MHz, Methanol-*d*_4_) δ ppm 3.91, 3.93 (4H, ABq, *J* = 17.4 Hz), 3.67 (3H, s), 3.47 (2H, s), 2.88 (4H, d, *J* = 17.4 Hz), 2.66 (4H, d, *J* = 17.4 Hz).

**^13^C NMR** (151 MHz, Methanol-*d*_4_) δ ppm 174.1, 161.1, 127.2, 72.3, 69.5, 59.8, 47.9, 34.9.

**HRMS (ESI-TOF^+^)***m/z*: calcd. for C_12_H_19_N_2_O_7_ [M + H]^+^: 303.1187, found: 303.1184.



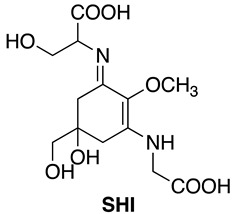



**SHI: ^1^H NMR** (600 MHz, Methanol-*d*_4_) δ ppm 4.22 (1 H, dd, *J* = 7.1, 3.8 Hz), 3.90–3.98 (3H, m), 3.80 (1H, dd, *J* = 11.5, 7.1 Hz), 3.70 (3H, s), 3.48, 3.45 (2H, ABq, *J* = 11.4 Hz), 2.98 (1H, d, *J* = 17.2 Hz), 2.89 (1H, d, *J* = 17.2 Hz), 2.72 (1H, d, *J* = 17.2 Hz), 2.65 (1H, dd, *J* = 17.2, 0.9 Hz).

**^13^C NMR** (151 MHz, Methanol-*d*_4_) δ ppm 174.4, 174.1, 161.3, 160.4, 127.3, 72.3, 69.5, 65.1, 62.1, 59.9, 47.9, 35.4, 34.8.

**HRMS (ESI-TOF^+^)***m/z*: calcd. for C_13_H_21_N_2_O_8_ [M + H]^+^: 333.1292, found: 333.1290.



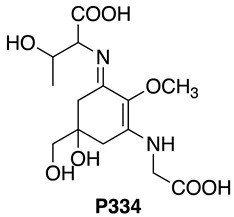



**P334: ^1^H NMR** (600 MHz, Methanol-*d*_4_) δ ppm 4.14 (1H, dt, *J* = 11.9, 6.1 Hz), 3.91-3.98 (3H, m), 3.70 (3H, s), 3.47, 3.45 (2H, ABq, *J* = 11.3 Hz), 2.93 (1H, d, *J* = 17.2 Hz), 2.89 (1H, d, *J* = 17.2 Hz), 2.67 (2H, m), 1.24 (3H, d, *J* = 6.4 Hz).

**^13^C NMR** (151 MHz, Methanol-*d*_4_) δ ppm 175.2, 174.1, 161.6, 160.4, 127.3, 72.3, 69.8, 69.5, 65.9, 60.0, 48.0, 35.2, 34.9, 21.0.

**HRMS (ESI-TOF^+^)***m/z*: calcd. for C_14_H_23_N_2_O_8_ [M + H]^+^: 347.1449, found: 347.1449.

**Figure 4:**
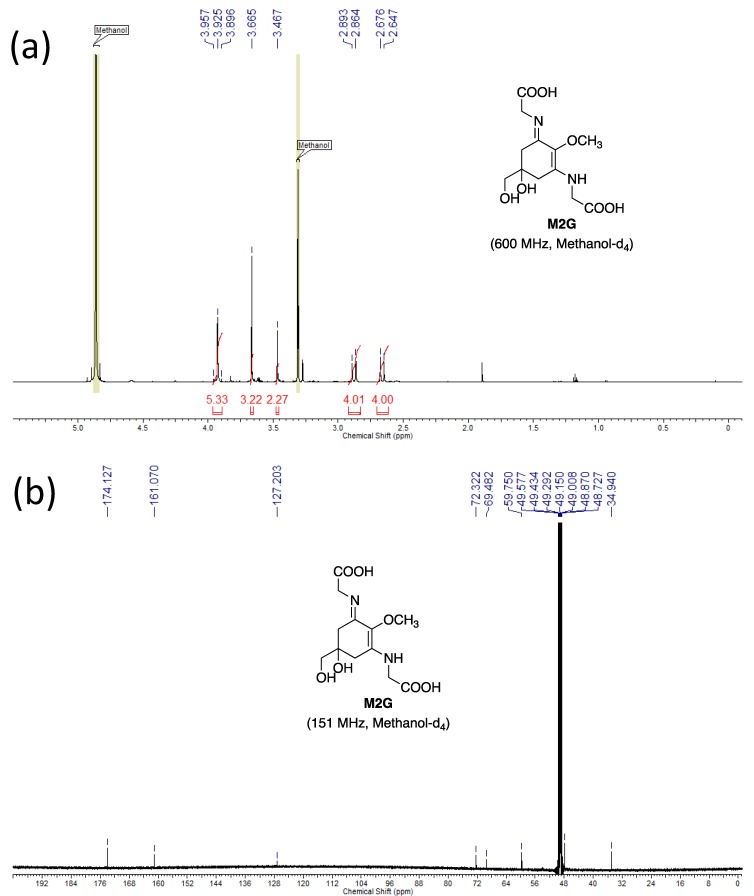
Nuclear magnetic resonance (NMR) spectra of M2G. (**a**) ^1^H NMR and (**b**) ^13^C NMR spectra are shown.

**Figure 5:**
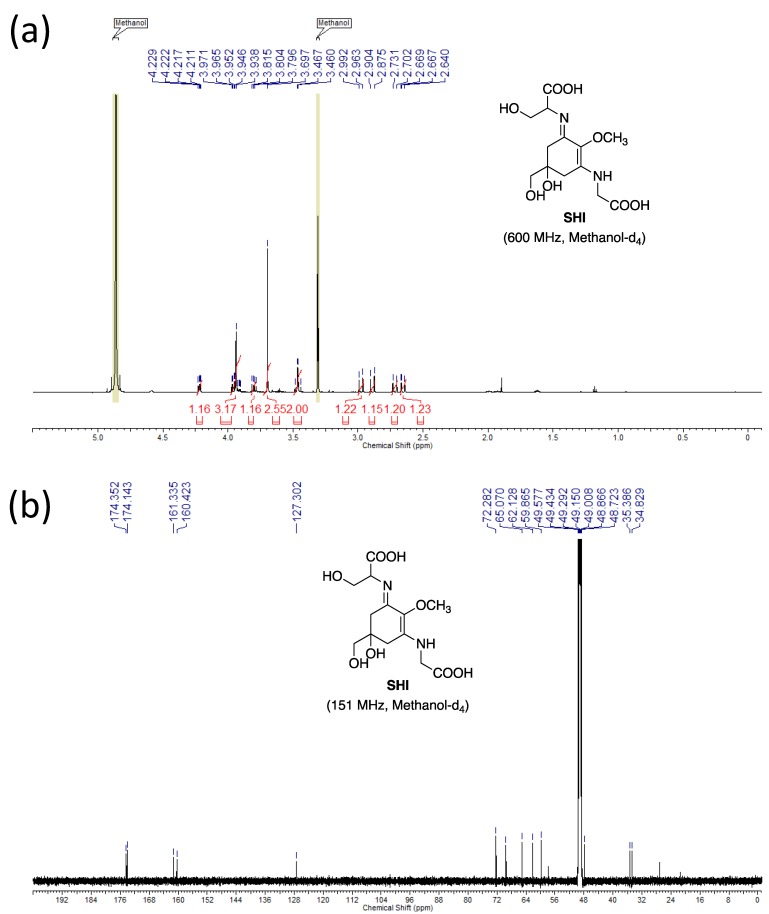
NMR spectra of SHI. (**a**) ^1^H NMR and (**b**) ^13^C NMR spectra are shown.

**Figure 6:**
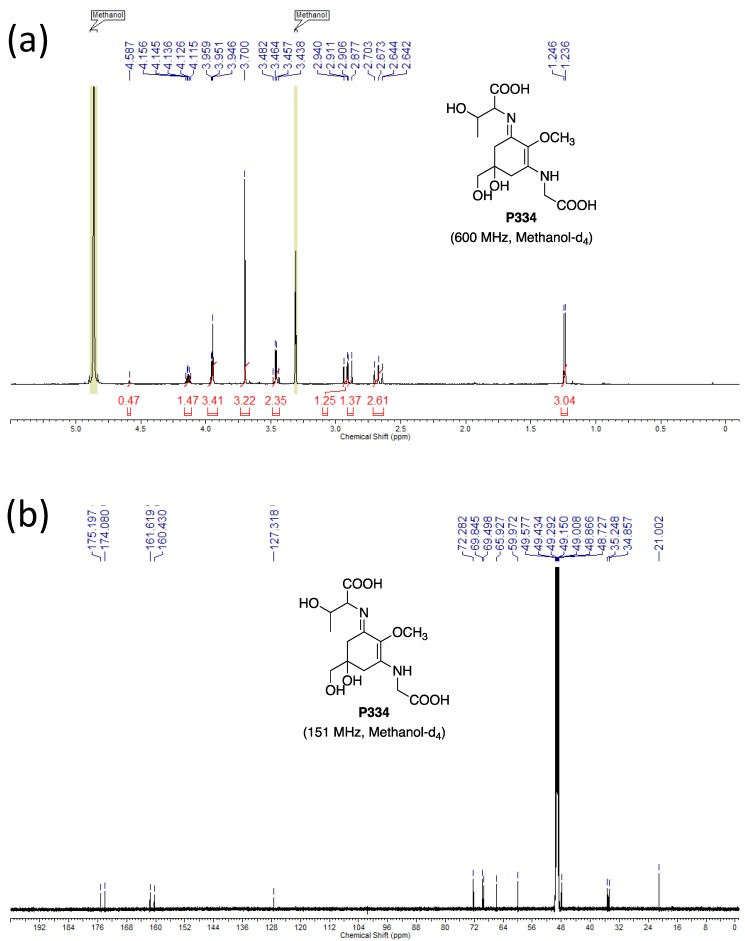
NMR spectra of P334. (**a**) ^1^H NMR and (**b**) ^13^C NMR spectra are shown.

The NMR spectra are shown in Figure 4, Figure 5 and Figure 6. The NMR spectra revealed that our purified MAA fractions contained low trace quantities of other organic compounds, such as components of the eluents. For example, the purified SHI reported previously contained trimethylamine, which was used for the eluent of preparative HPLC [16]. To the best of our knowledge, this is the first report showing the M2G NMR data recorded in methanol-*d*_4_.

To confirm whether the MAAs purified by our method are biologically active, we tested their antioxidant activities with an ABTS assay. The results indicated that each MAA possessed free radical scavenging activity (Table 2). The half maximal inhibitory concentration (IC_50_) values of SHI and P334 were comparable to the data published by another group [17]. On the other hand, M2G exhibited the strongest antioxidant activity amongst the MAAs analyzed in this study. This observation is consistent with our previous data using M2G and a mixture of P334 and SHI [4]. To the best of our knowledge, the data presented here is the first report that compared the free radical scavenging activity amongst these three MAAs, which were purified by the same laboratory. Thus, the MAAs obtained by our improved purification strategy were biologically active after purification; therefore, these materials can be further tested for various biological activities.

In conclusion, the method described above is broadly applicable for the isolation of MAAs from cyanobacteria. This method utilizing three-step liquid chromatographic separation may be suitable for the isolation of various types of MAAs.

In this method, after the extraction of the MAAs from cyanobacterial cells utilizing a simple methanol extraction protocol, the MAAs were then subjected to two-step reversed-phase liquid chromatography. Reversed-phase liquid chromatography is widely used because of its excellent separation characteristics, theoretical plate number, and reproducibility. It has been reported that 13 MAAs could be resolved with different retention times utilizing a classical analytical HPLC method based on a C18 column with a mobile phase containing acetic acid with isocratic elution [8]. However, the HPLC columns for preparative purification are large and expensive, and less costly methods are desirable. The particle size of the packing material is commonly 3–10 μm for analytical purposes, whereas larger particle sizes are often sufficient for preparative purposes. In our method, 40 μm was chosen as the most efficient particle size for separation, and an inexpensive general-purpose C18-type packing material was used. As this method required only small amounts of ethanol that functioned as the organic solvent during the separation step, polycarbonate chromatography columns could be used. Thus, the use of polycarbonate columns significantly reduced the cost of preparative purification. Moreover, purification on the same column using acidic eluent and neutral eluent efficiently eliminated impurities and greatly contributed to the cost-effectiveness of this technique.

In the third separation step utilizing gel filtration chromatography, water was used as an eluent. Therefore, after lyophilizing the purified product, the needless components of the eluent were removed. Therefore, the purified materials could be used with confidence in subsequent applications.

## Figures and Tables

**Table 1:** Purification yields of mycosporine-like amino acids (MAAs).

Purification Step	M2G ^1^		SHI ^2^		P334 ^2^	
	Amount (µg)	Yield (%)	Amount (µg)	Yield (%)	Amount (µg)	Yield (%)
Methanol extract or Chloroform extract	781	100	496	100	900	100
C18 (1% acetic acid)	498	63.8	427	86.1	748	83.1
C18 (0.1 M AcONH_4_)	357	45.7	260	52.4	358	39.8
SephadexG-10	288	36.9	227	45.8	322	35.8

^1^*A. halophytica* cells (3.3 g fresh weight) were used as starting material in this experiment. ^2^ 2.0 mL of Helioguard^®^365 was used as the starting material in this experiment.

**Table 2:** Free radical scavenging activity monitored by 2,2′-azino-*bis*(3-ethylbenzothiazoline-6-sulfonic acid) (ABTS) assay.

Compounds	IC_50_^1^ (µM)
M2G	40
SHI	94
P334	133
Trolox ^2^	10

^1^ Data shown represent the average of three independent experiments. ^2^ Trolox was used as a positive control. M2G—mycosporine-2-glycine; SHI—shinorine; P334—porphyra-334.
